# ESTIMATION OF PHOTON ENERGY AND DIRECTION DISTRIBUTIONS AT JAPANESE NUCLEAR POWER PLANTS BASED ON LITERATURE SURVEY FOR J-EPISODE STUDY

**DOI:** 10.1093/rpd/ncaa111

**Published:** 2020-09-03

**Authors:** Hiroshige Furuta, Akemi Nishide, Shin'ichi Kudo, Shin Saigusa

**Affiliations:** Institute of Radiation Epidemiology, Radiation Effects Association, 1-9-16 Kaji-cho, Chiyoda-ku, Tokyo 101-0044 Japan; Institute of Radiation Epidemiology, Radiation Effects Association, 1-9-16 Kaji-cho, Chiyoda-ku, Tokyo 101-0044 Japan; Institute of Radiation Epidemiology, Radiation Effects Association, 1-9-16 Kaji-cho, Chiyoda-ku, Tokyo 101-0044 Japan; Institute of Radiation Epidemiology, Radiation Effects Association, 1-9-16 Kaji-cho, Chiyoda-ku, Tokyo 101-0044 Japan

## Abstract

In order to reconstruct organ-absorbed dose from recorded dose for risk estimation in nuclear worker cohort, the preceding study of the International Agency for Research on Cancer (IARC) 15-Country Collaborative Study estimated the organ dose conversion factor from the recorded dose of *H*_p_(10) under the assumption that on average, in the nuclear power plants (NPPs), 10% of the dose received by workers was due to photon energies ranging from 100 to 300 keV and 90% from photon energies ranging from 300 to 3000 keV, with the average geometry being 50% in the antero-posterior geometry and 50% in the isotropic geometry. Similar examination was conducted at the Japanese Epidemiological Study on Low-Dose Radiation Effects (J-EPISODE).

Literature survey disclosed that Japanese electric power companies had jointly conducted the research on energy distribution and incidence direction distribution of gamma rays in working environments during periodical inspection and maintenance as well as during operation in the 1980s. The analysis of the survey results on photon energy and geometry distribution of Japanese NPPs demonstrated appropriateness in applying the IARC study assumption for nuclear workers in Japan and reconstructing organ-absorbed dose in the J-EPISODE. These results in Japan also provide strong evidence to support the robustness and generality of the IARC study assumption, which was estimated based on the judgment of experts at nuclear facilities around the world.

## INTRODUCTION

Although the concept of effective dose *E* and its operational definition of personal equivalent dose *H*_p_(10) are nowadays widely used for radiological protection purpose, the International Commission on Radiological Protection (ICRP) has recommended that effective dose should not be used for epidemiological studies^(1)^. It is desirable to use organ-absorbed dose for the evaluation of cancer morbidity and mortality in epidemiological cohort studies. Organ-absorbed dose was adopted for the International Agency for Research on Cancer (IARC) 15-Country Collaborative Study^(2–4)^, the International Nuclear Workers Study (INWORKS)^(5–8)^, Mayak study^(9)^ and the Life Span Study (LSS) of atomic bomb survivors^(10–12)^.

The IARC study assumption was that on average, in the nuclear power plants (NPPs), 10% of the dose received by nuclear workers was due to photon energies ranging from 100 to 300 keV and 90% from photon energies ranging from 300 to 3000 keV, and in the mixed activities (MA) facilities such as research and development organizations and fuel processing factories, 20% from photon energies ranging from 100 to 300 keV and 80% from photon energies ranging from 300 to 3000 keV, with the average geometry being 50% in the antero-posterior (AP) geometry and 50% in the isotropic (ISO) geometry for NPPs and MA facilities^(2)^, as shown in Table 1. Here, the exposure by nuclear workers was regarded to be derived from photon at energy level of 0.1–3 MeV and in AP geometry and ISO geometry, meaning that photon with energy over 3 MeV, exposure in rotational (ROT) geometry, neutron exposure and intakes of nuclides were thought to be negligible in the estimation of organ-absorbed dose. The IARC study determined the above-mentioned exposure condition basically based on the judgment of experts at nuclear facilities around the world, taking into consideration some prior experimental studies^(2)^.

**Table 1:** The assumption of the IARC study; estimated percentage of average doses in nuclear power plants and ‘MA’ facilities from different photon energies and different geometries of exposure.

Items	Percentage of dose received from different energy photons (keV)			Percentage of dose received in different geometries		
	0–100	100–300	300–3000	AP	Isotropic	Rotational
Nuclear power plants						
Average dose (%)	0	10	90	50	50	0
Range of dose (minimum–maximum) (%)	0–1	5–20	80–100	10–80	20–90	0
Uncertainty on average and ranges (%)	±5 (2 SD)			±10 (2 SD)		
‘MA’ facilities						
Average dose (%)	0	20	80	50	50	0

SD, standard deviation.

*Note:* Cited Table 4 in Thierry-Chef (2007)^(2)^ and reproduced by the author.

**Table 2:** Gamma-ray air dose rate and mean energy during PIM.

(1) PWR					
Survey spot	Location	Exposure rate (mR/H)			Mean gamma-ray energy (keV)
Ionization chamber	NaI^a^	
Spent fuel pit	A/B-5FL	2.4	A	1.6	653
Waste liquid evaporator room	A/B-3FL	14	A	17.4	1225
CVCS non-regeneration cooler room	A/B-3FL	14	A	8.6	851
RHR cooler room	A/B-2FL	8	A	2.5	836
RHR piping area A	A/B-1FL	26	A	5.2	920
RHR piping area B	A/B-1FL	14	A	3.2	845
RHR pump room	A/B-BFL	22	A	19.0	1113
5FL inside C/V	C/V-5FL	3.2	A	2.2	793
Loop room entrance	C/V-2FL	2.4	A	2.5	661
Beside the SG handhole	Loop room	80	D	32.7	685
Below the SG manhole	Loop room	36	B	28.7	877
Beside the SG barrier	Loop room	30	B	22.1	827
Pressurizer	Loop room	18	D	12.7	780
Reactor cooler pump	Loop room	30	D	4.0	767
(2) BWR					
Survey spot	Location	Exposure rate (mR/H)			Mean gamma-ray energy (keV)
		Ionization chamber	NaI^a^		
Condensate water filter room	T/B-1FL	0.0	C	0.05	859
Condensate demineralizer room	T/B-1FL	1.5	C	1.6	771
Radioactive waste disposal pump room	RW/B-1FL	2.5	A	4.3	992
Radioactive waste disposal tank room	RW/B-1FL	8.0	A	9.7	1097
Fuel inspection area	R/B-5FL	1.0	A	1.0	921
5FL inside R/B	R/B-5FL	4.0	A	2.6	382
Reactor well inside	R/B-5FL	9.0	A	8.3	1017
CRD repair room B	R/B-4FL	3.6	C	3.6	866
FPC heat exchanger room	R/B-3FL	4.8	A	5.4	807
CUW heat exchanger room	R/B-2FL	4.0	A	0.9	633
CUW auxiliary pump room	R/B-2FL	3.5	A	2.6	875
Around RHR pump	R/B-BFL	6.0	A	1.7	902
Equipment drain sump pump	R/B-BFL	3.5	A	4.4	989
Around feed-water nozzle	PCV-3FL	3.0	A	2.3	734
Around SRV A	PCV-2FL	18.0	A	15.9	1008
Around RHR/CUW piping	PCV-2FL	90.0	C	83.4	860
Around PLR ring header	PCV-2FL	25.0	C	11.5	736
Around PLR moter	PCV-1FL	9.0	A	6.9	1032
Around MSIV	PCV-1FL	6.0	A	4.7	879
Machine loading hatch front	PCV-1FL	11.0	A	7.3	1053
Pedestal inside	PCV-BFL	20.0	A	16.2	1312
Around floor drain sump pump	PCV-BFL	8.0	A	4.2	950

CVCS, chemical and volume control system; RHR, residual heat removal system; SG, steam generator; CRD, control rod drive; FPC, fuel pool cooling and cleanup system; CUW, reactor water cleanup system; SRV, safety relief valve; PLR, primary loop recirculation system; MSIV, main steam isolation valve.

^a^A: 3-inch spherical NaI, B: 2-inch spherical NaI, C: 1-inch spherical NaI, D: 1-inch diameter cylindrical NaI.

*Note*: Reproduced and translated by the author based on Figure 3.4.1 and Table 3.4.3 in REA (2019)^(21)^. The original was Figure A and Table 1 in EPCJCR (1983).^(19)^

**Table 3:** Gamma-ray direction during PIM.

(1) PWR										
Survey spot	Mean gamma-ray energy (keV)	Direction component (%) in Northern hemisphere								
		1. Top	2. Top front	3. Top left	4. Top back	5. Top right	6. Front	7. Left	8. Back	9. Right
Spent fuel pit	653	0	0	0	0	0	17	**47**	36	0
Waste liquid evaporator room	1225	0	18	0	0	0	**53**	15	14	0
CVCS non-regeneration cooler room	851	0	0	0	0	24	**63**	3	0	10
RHR cooler room	836	**37**	11	11	9	0	2	26	4	0
RHR piping area A	920	10	0	0	2	16	16	6	**44**	5
RHR piping area B	845	19	9	9	25	0	**34**	0	3	0
RHR pump room	1113	10	21	8	0	1	**51**	9	0	0
5FL inside C/V	793	13	**31**	16	3	0	25	13	0	0
Loop room entrance	661	15	**31**	9	7	0	29	0	2	7
Beside the SG handhole	685	0	2	0	0	0	**98**	0	0	0
Below the SG manhole	877	**78**	4	7	0	3	1	7	0	1
Beside the SG barrier	827	2	1	5	0	14	9	**45**	1	26
Pressurizer	780	0	**50**	4	15	0	9	1	21	0
Reactor cooler pump	767	—	—	—	—	—	—	—	—	—
(2) BWR										
Survey spot	Mean gamma-ray energy (keV)	Direction component (%) in northern hemisphere								
		1. Top	2. Top front	3. Top left	4. Top back	5. Top right	6. Front	7. Left	8. Back	9. Right
Condensate water filter room	859	0	0	0	0	0	-	0	0	-
Condensate demineralizer room	771	0	2	**76**	0	0	0	6	13	2
Waste disposal pump room	992	0	13	11	0	15	**59**	0	0	2
Waste disposal tank room	1097	0	**60**	5	1	4	21	6	0	4
Fuel inspection area	921	0	0	0	0	0	—	0	0	—
5FL inside R/B	382	4	15	0	0	0	22	22	**37**	0
Reactor well inside	1017	4	1	0	0	23	**45**	11	3	12
CRD repair room B	866	0	0	0	0	0	0	31	4	**65**
FPC heat exchanger room	807	13	17	3	13	10	**31**	0	5	9
CUW heat exchanger room	633	0	**53**	5	0	9	22	0	9	1
CUW auxiliary pump room	875	**42**	0	0	20	3	0	0	20	14
Around RHR pump	902	0	**44**	0	0	0	33	0	0	22
Equipment drain sump pump	989	12	0	10	19	19	0	10	**29**	0
Around feed-water nozzle	734	8	4	16	0	16	12	**44**	0	0
Around SRV A	1008	2	0	17	4	4	5	**47**	22	0
Around RHR/CUW piping	860	0	6	2	0	0	**56**	0	7	28
Around PLR ring header	736	2	0	5	0	1	11	**81**	0	0
Around PLR moter	1032	11	2	25	2	10	0	**36**	14	0
Around MSIV	879	0	25	4	0	0	**69**	1	0	1
Machine loading hatch front	1053	**42**	5	20	0	14	1	5	0	13
Pedestal inside	1312	0	**59**	0	23	12	2	3	0	3
Around floor drain sump pump	950	10	20	0	0	0	**61**	9	0	0

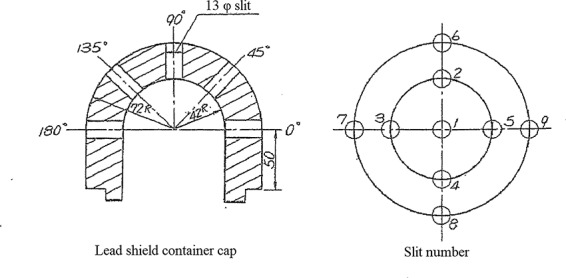

*Note*: (1) Reproduced and translated by the author based on Table 3.4.3 in REA (2019)^(21)^. The original was Figure A and Table 1 in EPCJCR (1983)^(19)^. (2) Unit of length: mm, φ: diameter, R: radius in the diagram at bottom left. The number in the direction component columns corresponded to that in the diagram at bottom right. (3) The values in bold represented the largest in respective rows.

Thierry-Chef *et al*. (2001)^(13)^ described a method to assess the proportion of the dose from photons in three energy ranges (<100, 100–300, ≥300 keV) using the responses under filters of a multi-element dosemeter and stated that the experimental, simulated data provided a good estimate of the proportion of dose from photons below 100 keV, the most critical for dosemeter response. Then the method was applied to personnel readings in one facility of Saclay, France, confirming the experts’ estimation. Thus, the expert’s estimation results of the IARC study assumption were supported by the experiment at Saclay. The authors^(13)^ also described that the results of the Saclay analyses were consistent with estimates of dose distribution with energy in the workplace carried out in the UK and USA.

### Japanese epidemiological study

The Japanese Epidemiological Study on Low-Dose Radiation Effects (J-EPISODE), funded by the Nuclear Regulation Authority (NRA), formerly by the Ministry of Education, Culture, Sports, Science and Technology (MEXT), has been conducted by the Radiation Effects Association (REA) since 1990 and analyzed health effects in association with radiation exposure evaluated in personal dose equivalent *H*_p_(10)^(14)^. However, in the above-mentioned, internationally evaluated radiation epidemiological studies, organ-absorbed dose is mainly used for the evaluation of morbidity and mortality due to cancer. In order for J-EPISODE to be compared and evaluated internationally in the future, it is indispensable to use organ-absorbed dose. In addition, cancer incidence data since 2016 have become available by the National Cancer Registry^(15)^. These conditions have enhanced J-EPISODE reconstruct organ-absorbed dose, and the Expert Committee on Reconstruction of Organ Dose was set up within REA during fiscal year 2017–2018.

### Aim of the study

The reconstruction of organ dose necessitates information on the photon energy and geometry distribution of the exposed population. The IARC study assumption seemed to be consistent with common knowledge based on the practical experience of radiation control staff in Japan. However, no document clearly stating working environment compatible with the IARC study assumption had been available in the public domain.

In order to verify the validity of the IARC study assumption also in Japan, a literature survey was conducted to review documents on working environment, such as photon energy distribution and geometry distribution of Japan’s NPPs, which was also pointed out by the Expert Committee. The present paper describes the results of a literature survey on energy distribution and geometry distribution in Japan’s NPPs and a supplementary analysis of the data. Reflecting the above result of literature survey, conversion factor from dosemeter reading to air kerma for nuclear worker for further conversion from air kerma to organ-absorbed dose was constructed in the preceding paper of Furuta *et al*. (2020)^(16)^.

## MATERIALS AND METHODS

The estimation of energy distribution and direction distribution of gamma ray was an important research item because it was the basic information for the evaluation of the personal dosemeter characteristics under actual working environment and the dose equivalent distribution in the body.

### Features of Japanese NPP

The reactor type of NPPs operated in Japan was a boiling water reactor (BWR) or a pressurized water reactor (PWR). There were 50 operating NPPs as of March 2013. Of these, 26 plants were BWR and 24 were PWR^(17)^. In Japan, periodical inspection by the successive regulatory authorities was implemented within at most 13 months from the previous one, and in many cases, nuclear operator conducted refueling, disassembling, maintenance and improvement work for dose reduction during shutdown period. Oumi *et al*. (2011)^(18)^ reported that the operation of the upper limit of regulation about 13 months and periodical inspection outage about 80 days were carried out in Japan and that the exposure dose during the periodical inspection usually contributed to 80–90% of the total annual dose. The authors^(18)^ also described the features of Japan’s working environment in comparison with foreign countries, especially the USA, as follows: the duration of plant operation was shorter, the duration of inspection activities was longer and the number of workers during outage was larger. In this connection, the term of ‘periodical inspection’ was hereafter referred to as ‘periodical inspection and maintenance (PIM)’.

### Disclosure of survey result

Regarding literature survey results, the 10 major electric power companies agreed to disclose the report of electric power companies’ joint commissioned research (hereafter called EPCJCR)^(19, 20)^ to REA and allowed REA to list the report as a reference and also to reproduce several tables and figures in the Expert Committee’s report, which was compiled in 2019, submitted as one of deliverables to NRA and then placed in the public domain^(21)^, in order to enable a sharing of the basic information of working environment with stakeholders, such as researchers, government officials and radiation control staff of NPP.

### Literature survey

During a literature survey, it was revealed that an energy spectrum analysis of the light-water reactor in Japan was actively conducted in the 1980s, when introduction of the concept of effective dose equivalent recommended by ICRP Publ. 26^(22)^ was considered. Since drastic changes were expected in the field of radiation protection at that time, it was considered that, in anticipation of the changes, proactive research activities had been carried out, which seemed also aggressive from a modern perspective.

**Figure 1:**
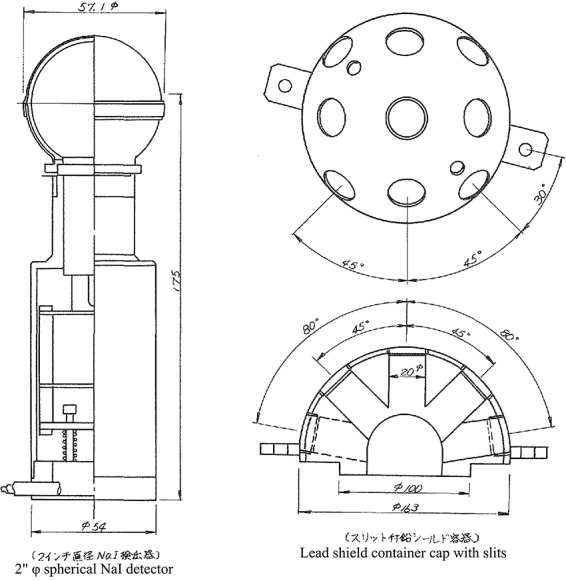
Example of gamma-ray direction measurement apparatus; NaI detector and lead shield container cap with slits. Cited Figure 1 in CRIEPI (1985)^(23)^. Unit of length: mm, φ: diameter

While conducting the literature survey, we arrived at the publicly accessible report of the Central Research Institute of Electric Power Industry (CRIEPI) (1985)^(23)^, which was a compilation of the result of technical studies on measurement method of direction distribution and energy distribution of radiation. With that as a clue, it was found that the 10 major electric power companies jointly conducted measurements on gamma-ray energy distribution and incident direction distribution at several NPPs in the 1980s, using the measurement method described in CRIEPI^(23)^. One of the EPCJCR was the survey during PIM^(19)^, the other was the survey during operation^(20)^. These two survey reports were disclosed to REA upon request.

However, the energy distribution during PIM described in EPCJCR (1983)^(19)^ was found only in the form of line charts of pulse-height count (PHC) data, the result of which could not be compared directly with the IARC study assumption. As a result of inquiring at each electric power company about the existence of investigation data on energy distribution during PIM, it was found that Tokyo Electric Power Company (TEPCO) holdings conserved the result of TEPCO commissioned survey during PIM^(24)^, which REA also applied for disclosure.

### EPCJCR survey method during PIM

Literature of EPCJCR (1983)^(19)^ investigated the energy distribution and incident direction distribution of gamma rays in the working area during PIM, exposure control during which was very important. As for the purpose of the survey^(19)^, the report described as follows: In line with the external exposure control of various workers at NPP, it was necessary to make an evaluation based on the determination of radiation field under the working environment. In 1981, the basic study on the related information and the preliminary test at the site of PWR were conducted, and in 1982, on-site evaluation of external exposure dose using the measurement apparatus was conducted on the main working areas during PIM at representative plants of PWR and BWR.

The work area investigated in the survey^(19)^ was 13 spots in PWR and 22 spots in BWR. In PWR, six spots in the reactor auxiliary building (A/B), two spots in the containment vessel (C/V) and five spots in the loop room were selected. In addition, in BWR, two spots in the turbine building (T/B), two spots in the radioactive waste disposal building (RW/B), nine spots in the reactor building (R/B) and nine spots in the primary containment vessel (PCV) were included in the survey. The report^(19)^ stated that the selected survey spots were well-represented because almost all the major works during PIM were performed there, or in the similar work environment.

### Measurement apparatus for energy distribution and direction distribution

In order to measure the energy distribution and direction distribution of gamma rays at NPP where the air dose rate spread widely, a measurement apparatus using NaI detector was manufactured^(19, 20, 23, 24)^. The apparatus was structured to be shielded by a lead material in order to reduce the influence of the background radiation, and a lead head portion with openable slits was provided on it so that gamma rays from a specific direction could be separated and measured. The slits of the lead container were provided at a total of nine positions in the northern hemisphere, at the north pole, at four equal division points on the 45° north circumference, and at four equal division points on the equator. Figure 1 is an example of a measurement apparatus using 2-inch spherical NaI^(23)^.

Regarding direction distribution, the spectral difference was measured by opening and closing the lead shielding plug of each slit. As for energy distribution, a PHC was measured without lead shield container cap on NaI detector. Then, the dose rate, the mean energy and the energy distribution of gamma rays could be calculated from this PHC data by applying the response matrix method. The NaI detector of 3-inch spherical type was mainly used, but in the high dose rate field, 2-inch spherical type or 1-inch spherical type was also used. For spectrum measurement, a portable pulse-height analyzer was employed^(23)^.

### TEPCO survey method during PIM

TEPCO conducted the survey on the radiation distribution of various working areas at Fukushima Daiichi Nuclear Power Station (BWR) in 1983–1984^(24)^. The survey work area was the whole area of Unit Three and the fifth floor of the R/B in Unit One.

The measurement method of the energy distribution of the gamma rays was the same as that of EPCJCR (1983)^(19)^. In addition to the PHC data, the analysis results of the gamma-ray intensity and the dose distribution by energy were listed in detail in tables and figures for each survey spot. Table of the cumulative dose contribution rate by energy at 100 keV band from 0 keV to 3000 keV was available for each survey spot. Thus, the value in the 200–300 keV band of the table indicated the proportion of dose <300 keV.

### EPCJCR survey method during operation

The EPCJCR (1986)^(20)^ implemented in 1984–1986 was intended to determine the dose rate distribution and energy distribution of high energy gamma rays in NPP during operation, when the radiation distribution status was regarded as different from that during PIM. Gamma-ray energy distribution was measured at a total of 19 spots in the selected two plants from BWR, and at a total of nine spots in the selected two plants from PWR. Although PWR survey measured inside C/V, BWR survey did not cover inside C/V because inert gas filled during operation. Direction distribution of gamma rays was not investigated.

## RESULTS

### EPCJCR survey result during PIM

Literature of EPCJCR (1983)^(19)^ described as follows: The main radiation type in each work area was gamma rays, and the exposure rate was at least 0.02 mR/H (ambient dose equivalent rate: 0.2 μSv/h) and at most 90 mR/H (0.9 mSv/h), as shown in Table 2. It was below 40 mR/H (0.4 mSv/h) in most work areas^(19, 21)^.

Although the main radiation source during PIM was ^60^Co (gamma-ray energies: 1.17 MeV and 1.33 MeV), the report^(19)^ stated that there were many work areas where the mean gamma-ray energy was in the range of 800–1000 keV due to the influence of scattering components, etc.

## Geometry distribution during PIM

According to Table 3, the actual working environment was not recognized as simple source distribution such as single point source. In many cases, the source direction in which the maintenance worker mainly exposed to radiation was found to be front or upper front.

Table 3 shows the results when the lead shield container with slits was left standing in the main source direction. In other words, it meant the exposure geometry when the worker was standing stationary at the maintenance spot toward the main source. The mean sum value of the column of ‘6. Front’ and ‘2. Top front’ of Table 3, which was assumed as AP geometry, was approximately 41% (interquartile range (IQR): 10–70%)^1^. The result supported that the proportion of AP was set up to an average of 50% and range 10–80% in IARC study assumption.

In addition to this, a video shooting of the actual worker’s motion during main jobs was implemented to analyze the worker’s ROT movements^(19)^. The survey stated that the worker’s movements showed the individuality such as right-handedness, fatigue, etc., and the rotation component was large even when the work was done in a specific direction. According to Table 4, the direction component of ‘Front’ by ROT motion analysis averaged 37%. It was found that even though the main source was located stationary in front of the measurement apparatus, the ROT movement of the actual worker contributed to increase the ratio of ROT or ISO in the exposure geometry. Considering that there were components of top front, top left, top back and top right in Table 2, it was appropriate for the worker’s exposure geometry to be regarded as ISO rather than ROT.

## Energy distribution during PIM

**Figure 2:**
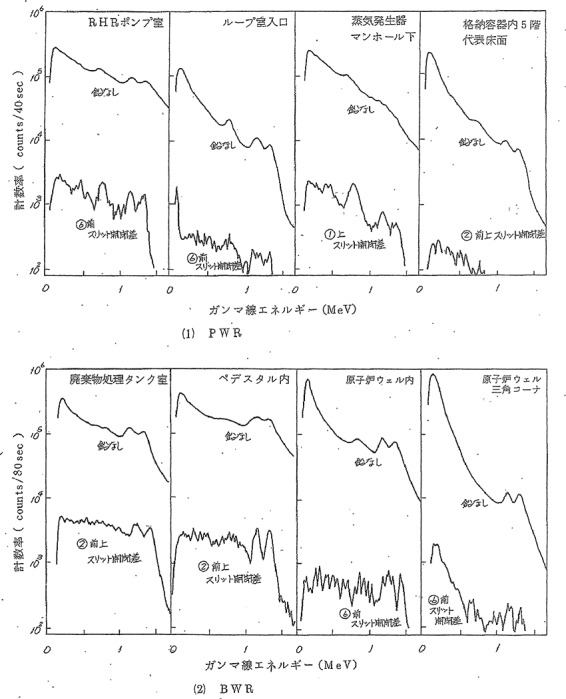
PHC of photon energy during PIM at PWR and BWR plant. Cited Figure 3.4.5 in REA (2019)^(21)^. The original was Figure 4 in EPCJCR (1983)^(19)^, written in Japanese. The caption was ‘Figure 4. Examples of measurement of gamma-ray energy spectrum’. The X-axis was ‘Gamma-rays energy (MeV)’, representing 0–1.5 MeV. The upper four line-chars were at PWR and the lower four at BWR. The Y-axis scale represented from 10^2^ to 10^6^. The unit of Y-axis at PWR was ‘Count rate (counts/40 sec)’ and that at BWR was ‘Count rate (counts/80 sec)’. Each chart had two lines, the upper of which was the measurement result without lead shield container cap on NaI detector, and the lower of which was the measurement result with the lead shielding plug of a specific slit being open. The survey spots at PWR were, from the left, RHR pump room, primary coolant loop room entrance, below the SG manhole and the fifth floor inside C/V, whereas those at BWR were radioactive waste disposal tank room, pedestal inside, reactor well inside and triangle corner in the reactor well.

Figure 2 displays an example of gamma-ray energy spectrum measurement for each of four representative working areas of PWR (upper) and BWR (lower) during PIM. The X-axis of each chart was gamma-ray energy (MeV) from 0 to 1.6 MeV, whereas the measurement was performed up to 3 MeV. The Y-axis was the common logarithm of the counting rate (PWR: counts/40 sec, BWR: counts/80 sec). That is, the figure represented gamma-ray PHC from NaI detector. However, neither a table nor a chart showing the energy distribution of the dose was included in the report^(19)^.

**Table 4:** Rotational movement analysis of real workers by video shooting during PIM.

Survey spot	Work description	Direction component when the phantom installation direction was front (%)							
		Front (0°)	Front right (45°)	Right (90°)	Back right (135°)	Back (180°)	Back left (225°)	Left (270°)	Front left (315°)
PWR									
Spent fuel pit	New fuel transportation	24	18	18	7	6	3	17	3
RHR pump room	Impeller installation	32	23	14	1	0	1	6	17
5FL inside C/V	Fuel support pin replacement	49	5	8	6	5	14	7	2
Beside the SG handhole	Lid closing	15	1	3	0	0	0	41	37
Below the SG manhole	Eddy current testing	40	17	11	2	2	1	9	15
Pressurizer	Inspection	45	3	1	0	1	2	16	28
Reactor cooler pump	Inspection	34	29	23	7	3	1	0	1
BWR									
Condensate water filter room	Filter out and transportation	5	10	7	8	13	33	10	11
Condensate demineralizer room	Inspection	23	10	13	1	7	0	15	27
Fuel inspection area	Refueling	48	12	10	0	0	0	11	16
5FL inside R/B	PCV head on	36	15	2	2	4	4	11	21
Reactor well inside	Decontamination	15	9	20	10	19	7	9	6
CRD repair room	Overhaul inspection	22^a^	15	8	7	9	9	9	16
Around SRV	Loading and assembly restoration	30^b^	5	1	1	3	3	12	42
RHR pump room	Inspection	68	10	9	0	1	2	3	4
PLR pump	Mechanical seal replacement	84	0	0	0	1	3	2	6
Pedestal inside	CRD recovery	60	8	9	7	0	0	1	12

^a^The direction of the tank was taken as front.

^b^The reactor tangential direction was taken as front.

*Note*: Reproduced and translated by the author based on Table 3.4.4 in REA (2019)^(21)^. The original was Table 6 in EPCJCR (1983)^(19)^.

### Trial estimation of energy distribution from PHC data

Although analysis method of PHC data were described in the Appendix in the present paper, the result was described here. Reading the line chart of counting rate of gamma rays at an interval of 0.1 MeV, the gamma-ray energy distribution under a certain assumption was calculated as shown in Table 5. The proportion of dose of 0.1–0.3 MeV was a mean of 11% of eight survey spots (IQR: 4–13%, mean for PWR: 9%, BWR: 14%) and was found to be within the range expected by the IARC study assumption.

### TEPCO survey result on gamma-ray energy distribution during PIM

**Figure 3:**
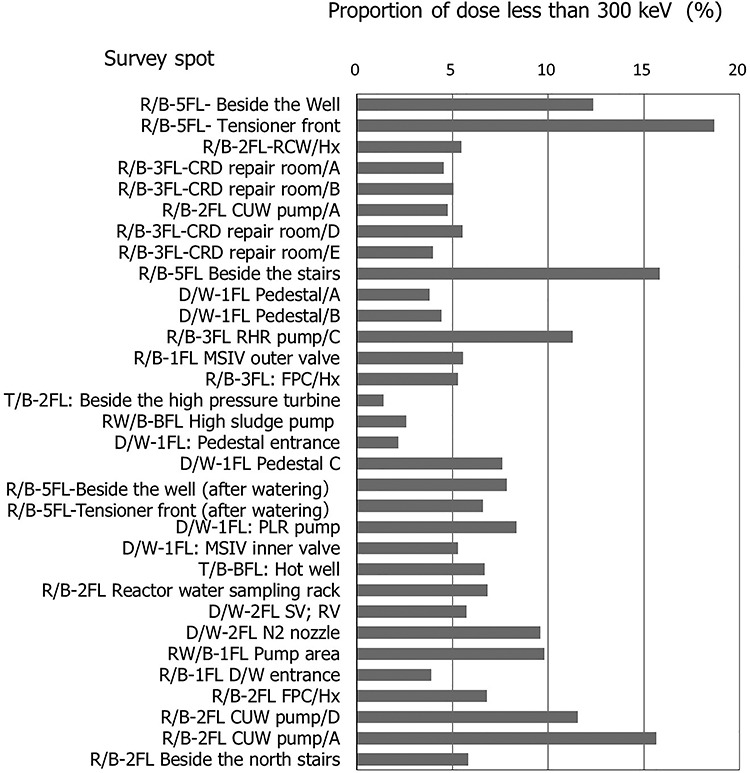
Gamma-ray energy distribution during PIM (TEPCO survey at Unit Three, Fukushima Daiichi Nuclear Power Station (BWR)). Cited Figure 3.4.7 in REA (2019)^(21)^ and translated by the author. The original data were on the tables in TEPCO (1984)^(24)^

Figure 3 shows the energy distribution at the 32 measurement spots in Unit Three, Fukushima Daiichi Nuclear Power Station except for the operation of PCV head off. The dose ratio of gamma rays with energy <300 keV showed the mean of 7.2% (IQR: 4.7–8.7%), and at most 18%, which were within the range envisioned by the IARC study assumption.

On the other hand, the energy distribution when opening PCV head was measured at Unit One, Fukushima Daiichi Nuclear Power Station. At the time of PCV head off operation, the energy distribution change of the gamma ray was expected. Making the use of high sensitivity type 3-inch height, 3-inch diameter cylindrical NaI detector, continuous measurement of energy distribution was performed. The dose ratio of <300 keV was summarized by stage when PCV head was opened, as shown in Table 6.

The dose ratio of <300 keV on the fifth floor of the R/B before opening PCV head was 13%, similar to the measurement results of Unit Three. As the opening work of the PCV head progressed, the mean energy value gradually decreased (not shown), whereas the dose ratio of <300 keV was at most 27%. The increased percentage of low energy gamma rays along with opening PCV head seemed to be due to the gamma rays from inside the reactor being scattered at the ceiling. When the well was filled full with water after completion of the PCV head off operation, the dose ratio of <300 keV returned to the value before the PCV head was opened.

**Table 5:** Trial estimation result of gamma-ray energy distribution during PIM.

Survey spot		Proportion of dose of 0.1–0.3 MeV (%)
PWR	RHR pump room	3.2
	Primary coolant loop room entrance	14.1
	Below the SG manhole	6.0
	The fifth floor inside C/V	13.2
	Mean	9
BWR	Radioactive waste disposal tank room	4.0
	Pedestal inside	2.7
	Reactor well inside	9.5
	Triangle corner in the reactor well	38.4
	Mean	14
Mean of the eight survey spots		11

*Note*: Cited Table 3.4.6 in REA (2019)^(21)^ and translated by the author. The estimation method was described in the Appendix in the present paper.

**Table 6:** Change of gamma-ray energy distribution during PCV head off (TEPCO survey) (beside the reactor well on the fifth floor in the R/B, Unit One, Fukushima Daiichi Nuclear Power Station (BWR)).

Stages when opening PCV head	Proportion of dose <300 keV (%)
Before PCV head opening	(Seven-spot mean) 12.5
The day before PCV head opening	7.6
Just before opening the PCV head	13.2
Lifting the PCV head in the reactor well	20.3
Putting the PCV head on the fifth floor	21.1
PCV head opened completely	27.4
Reactor well filled with water after moving the dryer	(Two-spot mean) 11.0

*Note*: Cited Table 3.4.8 in REA (2019)^(21)^ and translated by the author. The original data were on the tables in TEPCO (1984)^(24)^.

### EPCJCR survey result during operation in BWR

In the BWR during operation, there was a spot where gamma rays with much higher energy than ^60^Co were generated. The nuclides of ^16^N (half-life: 7.13 sec) and ^15^C (half-life: 2.4 sec) were found which emit high energy gamma rays (^16^N: 6.1 MeV and ^15^C: 5.3 MeV) while moving along the steam flow^(20)^ However, it was concluded that the effects of high energy gamma rays from ^16^N and ^15^C were negligible during PIM, because such nuclides disappeared shortly after shutdown due to their short half-lives and were no more produced during PIM.

**Figure 4:**
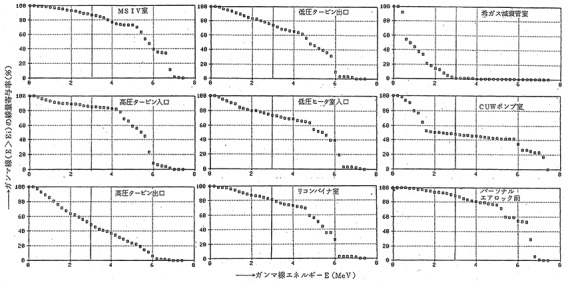
Gamma-ray energy distribution during operation at BWR plant. Cited Figure 3.4.9 in REA (2019)^(21)^. The original was Figure 3 in EPCJCR (1986)^(20)^, written in Japanese. The caption was ‘Figure 3. Dose contribution rate of each energy of gamma rays at selected survey spots in Hamaoka Nuclear Power Plant (BWR)’. The X-axis was ‘Gamma-rays energy E (MeV)’ and the Y-axis was ‘Dose contribution rate of each energy of gamma rays (E > Ei); %’. The nine line-charts displayed respective dose contribution of survey spots; from upper left to right, MSIV room, low pressure turbine room exit, rare gas hold-up pipe room, high pressure turbine room entrance, low pressure heater room entrance, CUW pump room, high pressure turbine room exit, recombiner room and front of personal air lock.

Figure 4 displays the dose contribution rates by gamma-ray energy at the nine survey spots in Hamaoka Nuclear Power Station (BWR). The X-axis of each chart was gamma-ray energy (MeV), which was displayed 0–8 MeV at 0.2 MeV bands. The Y-axis represented the cumulative dose contribution rate of gamma rays above the energy at the indicated point. Therefore, assuming that the contribution rates of the 0.1 MeV point, the 0.3 MeV point and the 3 MeV point were a, b and c, respectively, the ratio of (a − b) and (b − c) meant the dose ratio of 0.1–0.3 MeV and 0.3–3 MeV.

According to the results of reading the charts, the proportion of dose of 0.1–0.3 MeV was in the range of 4 to 10% (Mean 7%), indicating that the results were within an envisioned range of IARC study.

### EPCJCR survey result during operation in PWR

During the operation of PWR, high-energy gamma rays from ^16^N and ^15^C were also measured. In addition, gamma rays with energy about 8 MeV, which was higher than that of ^16^N, were also detected. It was considered that such gamma rays were emitted from the ^56^Fe (n, γ) ^57^Fe neutron capture reaction, in case that ^56^Fe were contained in the reactor structural material and captured neutrons generated by fission of ^235^U. Therefore, such high gamma rays were observed only inside C/V and in front of its emergency air lock near the reactor^(20)^.

**Table 7:** Gamma-ray direction distribution based on the EPCJCR.

Proportion of AP geometry (%)	During PIM		During operation		Assumption of IARC study
	BWR (22 spots)	PWR (13 spots)	BWR	PWR	
Mean	39	46	Not surveyed		50
IQR	4–72	16–62			—
Min—Max	0–94	5–100			10–80

*Note*: (1) The above figures based on the results when the lead shield container with slits was left standing in the main source direction. (2) Cited Table 3.4.11 in REA (2019)^(21)^ and translated by the author.

**Table 8:** Gamma-ray energy distribution based on the EPCJCR.

Proportion of dose in 0.1–0.3 MeV (%)	During PIM		During operation		Assumption of IARC study
	BWR (4 spots)	PWR (4 spots)	BWR (9 spots)	PWR (5 spots)	
Mean	14	9	7	10	10
IQR	4–17	5–13	5–8	10–11	—
Min—Max	3–38	3–14	4–10	3–15	5–20

*Note*: Cited Table 3.4.12 in REA (2019)^(21)^ and translated by the author.

**Figure 5:**
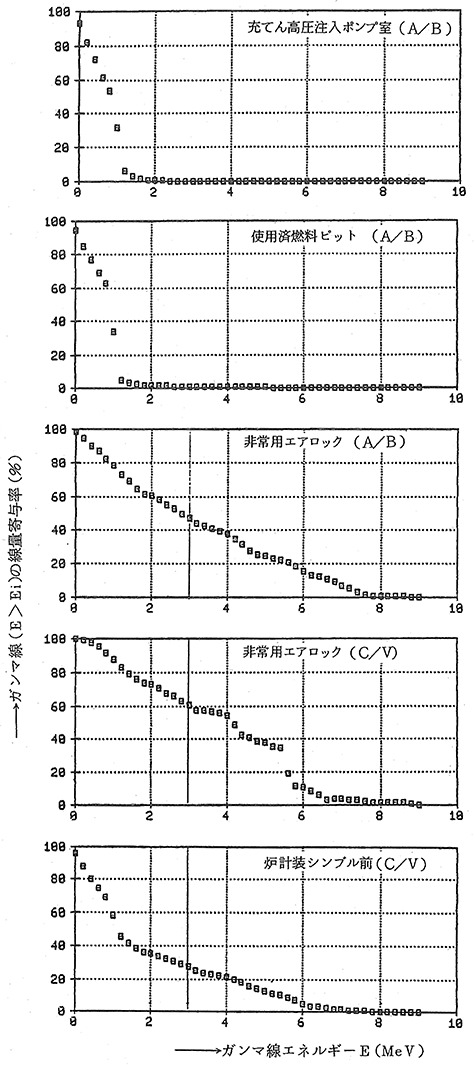
Gamma-ray energy distribution during operation at PWR plant. Cited Figure 3.4.10 in REA (2019)^(21)^. The original was Figure 5 in EPCJCR (1986)^(20)^, written in Japanese. The caption was ‘Figure 5. Dose contribution rate of each energy of gamma rays at selected survey spots in Mihama Power Plant (PWR)’. The X-axis was ‘Gamma-rays energy E (MeV)’ and the Y-axis was ‘Dose contribution rate of each energy of gamma rays (E > Ei); %’. The five line-charts displayed respective dose contribution at survey spots; from upper to bottom, charging/safety injection pump room (A/B), spent fuel pit (A/B), emergency air lock (A/B), emergency air lock (C/V) and front of the in-core neutron monitoring system thimble tubes (C/V).

Figure 5 displays the dose contribution rates by energy of gamma rays at five survey spots in Mihama Power Station (PWR). Similar to the above Figure 4 of BWR, but the X-axis was displayed from 0 to 9 MeV at 0.2 MeV bands. The estimated proportion of dose 0.1–0.3 MeV ranged from 3 to 15% (mean 10%). The result in PWR during operation was also found to be almost within an envisioned range of IARC study.

### Summary

With regard to the gamma-ray incident direction distribution during PIM in Table 7, the AP component did not reach 50% with the stationary measurement apparatus. However, considering the movement of the actual worker by video shooting, the AP ratio of the exposure geometry was not considered inconsistent with the IARC study assumption.

Despite a lack of data during operation, the direction distribution during operation was understood that it was the same as those during PIM.

Table 8 summarizes the energy distribution of gamma rays at Japanese NPP^(21)^. The proportion of dose in 100–300 keV was found to be within an envisioned range of IARC study.

In the end, it was verified that the surveys were conducted to assess the energy spectrum and directions of incidence on workers and that those surveys demonstrate that both the spectrum and the direction of incidence measured are compatible with the IARC study assumption.

## DISCUSSIONS

### Estimation method of energy distribution

The approach for estimating energy distribution differed between Thierry-Chef (2001)^(13)^ and EPCJCR^(19, 20)^. The former used the differences of the responses under filters of a multi-element dosemeter, which was worn by a worker, whereas the latter used PHC data from a NaI detector, which was placed stationary at a specific working area. Despite the different approaches, it was interesting that the both resulted in almost the same ratio of energy 100–300 keV and 300–3000 keV.

### Trial estimation of energy distribution from PHC data

The gamma-ray energy distribution during PIM at TEPCO whose energy distribution was not based on the trial estimation, but derived directly from the tables listed in the survey report^(24)^ was evaluated as being within an envisioned range of IARC study. In addition, the result of the trial estimation (Table 5) was almost the same as the result of TEPCO (Figure 3). Therefore, the trial method of estimating dose distribution by energy from PHC data was confirmed to be appropriate.

### Gamma rays above 3 MeV during operation

Figures 4 and 5 show that a considerable portion of gamma rays >3 MeV was present during operation. However, since only a limited number of operators and radiation control staff entered the control area during operation, and the stay inside the control area was also in short time, the portion of exposed gamma rays >3 MeV was considered small.

### Representativeness of the survey result over a long period

J-EPISODE has published analysis reports every 5 y since 1995. The fifth analysis of J-EPISODE targeted the dose data during 1957–2010, at an intermediate point of which the EPCJCR were conducted. Although the dose rate had dropped sharply in the 1980s as a result of the dose reduction measures, the reactor type, the radiation source and job description basically remained without a big change, suggesting that the survey results in the 1980s represented the whole period 1957–2010. That is, it turned out that there was appropriate in applying the energy and geometry assumption of IARC study to J-EPISODE.

## CONCLUSIONS

The IARC study assumption on the energy and geometry distribution of photon exposure is fundamental information in estimating organ-absorbed dose from personal dosemeter reading. Verifying whether the IARC study assumption can be applied to Japanese workers in NPP will be crucial for the assessment of reconstructed organ-absorbed dose.

As a result of the literature survey in the 1980s, it was found that there were reports of investigation by EPCJCR where electric power companies actually measured and assessed the energy spectrum and directions of incidence on workers at the site of NPP separately during PIM and during operation. The results of EPCJCR became the evidence that the IARC study assumption was applicable for J-EPISODE. The analysis of working environment of Japanese workers in NPP demonstrated appropriateness in applying the IARC study assumption for reconstructing organ-absorbed dose in J-EPISODE. These results in Japan also provide strong evidence to support the robustness and generality of the IARC study assumption, which was estimated based on the judgment of experts at nuclear facilities around the world.

## Footnotes

1 One survey spot of reactor cooler pump in PWR and two spots of condensate water filter room and fuel inspection area in BWR were excluded because of no description of the results.

## ABBREVIATIONS DEFINITIONS

A/B, auxiliary building; BWR, boiling water reactor; C/V, containment vessel; CRD, control rod drive; CUW, reactor water cleanup system; CVCS, chemical and volume control system; D/W, dry well; FPC, fuel pool cooling and cleanup system; MSIV, main steam isolation valve; PCV, primary containment vessel; PLR, primary loop recirculation system; PWR, pressurized water reactor; R/B, reactor building; RCW, reactor building closed cooling water system; RHR, residual heat removal system; RW/B, radioactive waste disposal building; SG, steam generator; SRV, safety relief valve; T/B, turbine building.

## NOTE

The above list was based on the Information Portal for the Fukushima Daiichi Accident Analysis and Decommissioning Activities. Available from: https://fdada.info/en/home2/abbrev_top2/abbrev_search-en/ (5 July 2020, date last accessed).

## Appendix. Trial estimation method of energy distribution of gamma ray from PHC of NaI detector

A case of ‘below the steam generator (SG) manhole’ of PWR is shown below.

(1) From the line of ‘without lead shield container cap on NaI detector’ at ‘below the SG manhole’ in Figure 2, the PHC between 0.1 and 1.6 MeV at 0.1 MeV interval was digitized as Table A1.

**Table A1:** PHC of ‘below the SG manhole’.

Energy band (MeV)															
0.1	0.2	0.3	0.4	0.5	0.6	0.7	0.8	0.9	1.0	1.1	1.2	1.3	1.4	1.5	1.6
Counting rate (1000 counts/40 sec)															
77	251	194	158	123	105	105	74	57	46	42	36	26	18	12	9

**Table A2:** Compton scattering fraction ratio.

0.2	0.10	1.00													
0.3	0.21	0.08	1.00												
0.4	0.27	0.22	0.03	1.00											
0.5	0.29	0.23	0.22	0.01	1.00										
0.6	0.23	0.23	0.22	0.25	0.02	1.00									
0.7	0.23	0.23	0.23	0.27	0.22	0.02	1.00								
0.8	0.21	0.21	0.21	0.24	0.28	0.20	0.02	1.00							
0.9	0.21	0.21	0.21	0.22	0.24	0.28	0.20	0.04	1.00						
1.0	0.21	0.21	0.21	0.21	0.22	0.23	0.29	0.20	0.05	1.00					
1.1	0.20	0.20	0.20	0.21	0.21	0.22	0.27	0.26	0.18	0.06	1.00				
1.2	0.20	0.20	0.20	0.20	0.21	0.22	0.22	0.28	0.29	0.17	0.07	1.00			
1.3	0.19	0.19	0.21	0.21	0.21	0.21	0.26	0.26	0.32	0.35	0.18	0.25	1.00		
1.4	0.19	0.19	0.19	0.20	0.22	0.22	0.22	0.26	0.29	0.34	0.38	0.18	0.29	1.00	
1.5	0.18	0.18	0.19	0.19	0.19	0.21	0.22	0.23	0.25	0.29	0.33	0.40	0.17	0.27	1.00
1.6	0.19	0.19	0.19	0.19	0.19	0.20	0.20	0.19	0.24	0.27	0.31	0.34	0.41	0.19	0.23	1.00
MeV	0.1	0.2	0.3	0.4	0.5	0.6	0.7	0.8	0.9	1.0	1.1	1.2	1.3	1.4	1.5	1.6

Considering the Compton scattering fraction in the PHC, the number of incident photons by energy, called as unfolded data or corrected count data, was estimated as follows.

(2) The counting rate of 9 (1000 counts/40 sec) at the energy band of 1.6 MeV, indicating a width of 1.5–1.6 MeV, was assumed to be derived from gamma ray of that energy band.

(3) Compton scattering fraction at lower energy bands derived from the highest energy of 1.6 MeV was firstly removed from the PHC, as the next. Figure 2 (4) of the response matrix (0–3 MeV) for 3-inch spherical NaI in the reference of CRIEPI (1985)^(23)^ was used for the estimation of the Compton scattering fraction. It was assumed that the response matrix was M(i, j) (i, j = 1, 2,..., 30), i represented an gamma-ray incident energy band of i/10 (MeV), j represented channel of energy band j/10 (MeV) and M(i, j) indicated the PHC at channel of energy band j/10 (MeV) in case of gamma-ray incident with energy band of i/10 (MeV). The Compton scattering fraction ratio derived from gamma-ray incident of 1.6 MeV (i = 16) was defined as M(16, j)/M(16, 16) for channel of the lower energy band j (j = 1, 2,..., 15). For each incident energy band (the i-th row), the Compton scattering fraction ratio included in the lower energy band (the j-th column) is determined as Table A2.

(4) The PHC of 9 at 1.6 MeV (Table A1) was multiplied by the Compton scattering fraction ratio at the energy band j of the 1.6 MeV row (i = 16) in Table A2, followed by subtracting the multiplication value from PHC at the energy band j in Table A1.

Then, with respect to 1.5 MeV energy band, next to the highest energy band, the same process described above was performed. The steps were sequentially performed until the lowest energy band, completing the corrected count data. This process, called stripping method, was illustrated as the triangular matrix (Table A3). A solution could not be obtained because the PHC of 0.1 MeV or less disappeared for the energy of 0.4 MeV or less. The reason might be that there was no information on the lower limit energy of the pulse height and that the response matrix itself was a reference value. However, since this was a simple analysis, the process was repeated until peak components could be subtracted.

**Table A3:** Corrected count data (1000 counts/40 sec).

MeV	0.1	0.2	0.3	0.4	0.5	0.6	0.7	0.8	0.9	1.0	1.1	1.3	1.3	1.4	1.5	1.6
1.6	77	251	194	158	123	105	105	74	57	46	42	36	26	18	12	9
1.5	75	249	192	156	121	103	103	72	55	44	39	33	22	16	10	
1.4	74	248	190	154	119	101	101	70	52	41	36	29	21	14		
1.3	71	245	188	152	116	98	98	66	48	36	31	27	17			
1.2	68	242	184	148	113	95	94	62	43	30	28	22			
1.1	63	237	180	144	108	90	89	56	36	26	26				
1.0	58	232	175	138	103	84	82	49	32	25					
0.9	53	227	169	133	97	78	74	44	30						
0.8	47	221	163	126	90	70	68	43							
0.7	37	211	154	116	78	61	67								
0.6	22	196	138	98	63	59									
0.5	8	182	125	83	62										
0.4	0	168	111	82											
0.3	0	150	109												
0.2	0	142													
Unfold	0	142	109	82	62	59	67	43	30	25	26	22	17	14	10	9

**Table A4:** Gamma-ray flux by energy band.

Energy band (MeV)															
0.1	0.2	0.3	0.4	0.5	0.6	0.7	0.8	0.9	1.0	1.1	1.2	1.3	1.4	1.5	1.6
Gamma-ray flux (unit: 10^−3^ γ/cm^2^/sec)															
0	54	53	49	47	52	69	50	39	35	38	35	28	25	19	19

**Table A5:** Air kerma rate by energy band.

Energy band (MeV)															
0.1	0.2	0.3	0.4	0.5	0.6	0.7	0.8	0.9	1.0	1.1	1.2	1.3	1.4	1.5	1.6
Air kerma rate (unit: 10^−15^ Gy/h)															
0	50	74	91	110	144	218	180	156	154	182	181	154	144	118	123

The first row at 1.6 MeV in Table A3 came from Table A1. When the incident of gamma ray at 1.6 MeV was ‘9’, the value at 1.5 MeV row was obtained by subtracting the Compton scattering fraction generated thereby from PHC of the first row at the energy band <1.5 MeV (j = 1, 2,..., 15). Next, when ‘10’ of the 1.5 MeV band in column of the 1.5 MeV row was incident, the Compton scattering fraction generated thereby was subtracted from the corrected PHC at energy band of 1.4 MeV or less. This was repeated until the 0.2 MeV row. The lowermost row of ‘unfold’ was obtained by taking out diagonal components of a triangular matrix and was the corrected count data in which Compton scattering fraction had been removed (unit: 1000 counts/40 sec).

(5) The corrected count data were converted into gamma-ray flux by energy band in consideration of detection sensitivity of NaI detector, as shown in Table A4. For detection sensitivity, the Figure 7.3 of ‘gamma counting efficiency (%) of NaI (Tl) scintillation detector’ in MEXT (1974)^(25)^ was referred.

(6) The gamma ray flux by energy band was converted to air kerma rate (unit: 10^−15^ Gy/h), as shown in Table A5. The fluence–kerma conversion factor (unit: 10^−12^ Gy cm^2^) was applied by interpolating Table A.1 of ICRP Publ. 74^(26)^ with quadratic formula. Based on this air kerma rate, the dose ratio of <0.3 MeV was estimated as the next.

Less than 0.3 MeV:0.3 MeV and over = 124: 1954 = 6%: 94%.
